# Cardiac Tissue Engineering: Inclusion of Non-cardiomyocytes for Enhanced Features

**DOI:** 10.3389/fcell.2021.653127

**Published:** 2021-05-25

**Authors:** Sadek Munawar, Irene C. Turnbull

**Affiliations:** Cardiovascular Research Center, Icahn School of Medicine at Mount Sinai, New York, NY, United States

**Keywords:** engineered cardiac tissues, 3D physiological models, cardiomyocytes, nonmyocytes, cell-cell interactions, fibroblasts, endothelial cells, human induced pluripotent stem cell-derived cardiomyocytes

## Abstract

Engineered cardiac tissues (ECTs) are 3D physiological models of the heart that are created and studied for their potential role in developing therapies of cardiovascular diseases and testing cardio toxicity of drugs. Recreating the microenvironment of the native myocardium *in vitro* mainly involves the use of cardiomyocytes. However, ECTs with only cardiomyocytes (CM-only) often perform poorly and are less similar to the native myocardium compared to ECTs constructed from co-culture of cardiomyocytes and nonmyocytes. One important goal of co-culture tissues is to mimic the native heart’s cellular composition, which can result in better tissue function and maturity. In this review, we investigate the role of nonmyocytes in ECTs and discuss the mechanisms behind the contributions of nonmyocytes in enhancement of ECT features.

## Introduction

Cardiovascular disease is the leading cause of death in the United States (Benjamin et al., 2019). Despite improvements in acute treatment of cardiovascular disease, the incidence of heart failure is increasing (Cahill and Kharbanda, 2017; Lesyuk et al., 2018). Available therapies for heart failure include invasive procedures such as heart transplant or the implantation of a mechanical assist device as a bridge to transplant; non-invasive therapies include drugs aimed at improving contractility (Gheorghiade et al., 2016). Meanwhile, alternative therapies are being investigated to repair and regenerate the damaged myocardium to restitute its function; these alternative therapies under investigation include small molecules, growth factors, gene (lentiviral, adenoviral, and adeno-associated viral vectors (AAVs) and cell therapies (Talman and Kivelä, 2018; Kieserman et al., 2019). Challenges with the clinical translation of novel therapies for the heart indicate an incomplete understanding of the underlying biological mechanisms involved. Tissue engineering technologies are emerging as a method to overcome these hurdles and develop human-specific models of the myocardium. Engineered tissues may allow mechanistic study of the cellular interactions that enhance cardiac function (Bursac et al., 2010). Human engineered cardiac tissues (hECTs) can serve to bridge the gap between current animal models, providing a species-specific model of human myocardium, and also overcomes limitations of the 2D culture systems (Vunjak Novakovic et al., 2014; Greenberg et al., 2018). Human pluripotent stem cells now provide a nearly limitless supply of differentiated human cardiomyocytes (CMs) (Lian et al., 2012; Bhattacharya et al., 2014), and use of these cells to create 3-D hECTs allows direct measurement of twitch force and related characteristics of cardiac muscle contractility, with extended time in culture (Turnbull et al., 2014).

There has been a rapid growth in the field of tissue engineering with constant improvements for the fabrication of a structural and functional mature model of human myocardium (Ruan et al., 2015; Feric and Radisic, 2016; Shadrin et al., 2017; van den Akker et al., 2018; Ronaldson-Bouchard et al., 2019). Their utility as *in vitro* test tools have been demonstrated by their application for drug screening (Feric et al., 2019; Keung et al., 2019). However, the engineered cardiac tissues (ECTs) are immature and mostly resemble the fetal heart. While techniques such as electrical stimulation, mechanical stress, and longer time in culture improve tissue maturity, ECTs are not as mature as adult myocardium (Mahowald et al., 2009; Nunes et al., 2013; Schwach and Passier, 2019). Human induced-pluripotent stem cell (hiPSC)-derived CMs are widely used in cardiac tissue engineering. However, these cells have immature fetal-like characteristics in terms of gene expression, sarcomere organization, force of contraction, and action potentials (Yang et al., 2014; Veerman et al., 2015). Most of the technologies in cardiac tissue engineering have involved the use of CMs (from neonatal rat hearts or derived from pluripotent stem cells), with little or no contribution of other nonmyocyte cells. While these approaches have contributed to great advances in the field, the contribution of nonmyocytes requires more attention. Understanding the intercellular signaling within the myocardium is a relevant aspect in the development of new therapies with impact on cardiac function.

Incorporating non-CMs can make the cellular composition of the tissues better mimic natural myocardium. The heart is composed of multiple cell types. The reported cellular composition differs based on the methods used to identify the cell types and quantify their abundance. Using flow cytometry and immunohistochemical analysis, Pinto et al. (2016) found that around 31% of cells in a mouse heart are CMs; the non-CM population of the heart includes approximately 60% endothelial cells (ECs), 5–10% hematopoietic-derived cells, and fibroblasts (FBs) under 20%. Furthermore, using single nuclear RNA-sequencing of samples from human donors, Tucker et al. (2020) identified nine major cell types with CMs making up 35.9% of the total population. An intricate network of interactions between the distinct non-CM cell types and CMs support cardiac homeostasis (Hirsch et al., 2014; Dunn and Palecek, 2018; Colliva et al., 2020).

**FIGURE 1:**
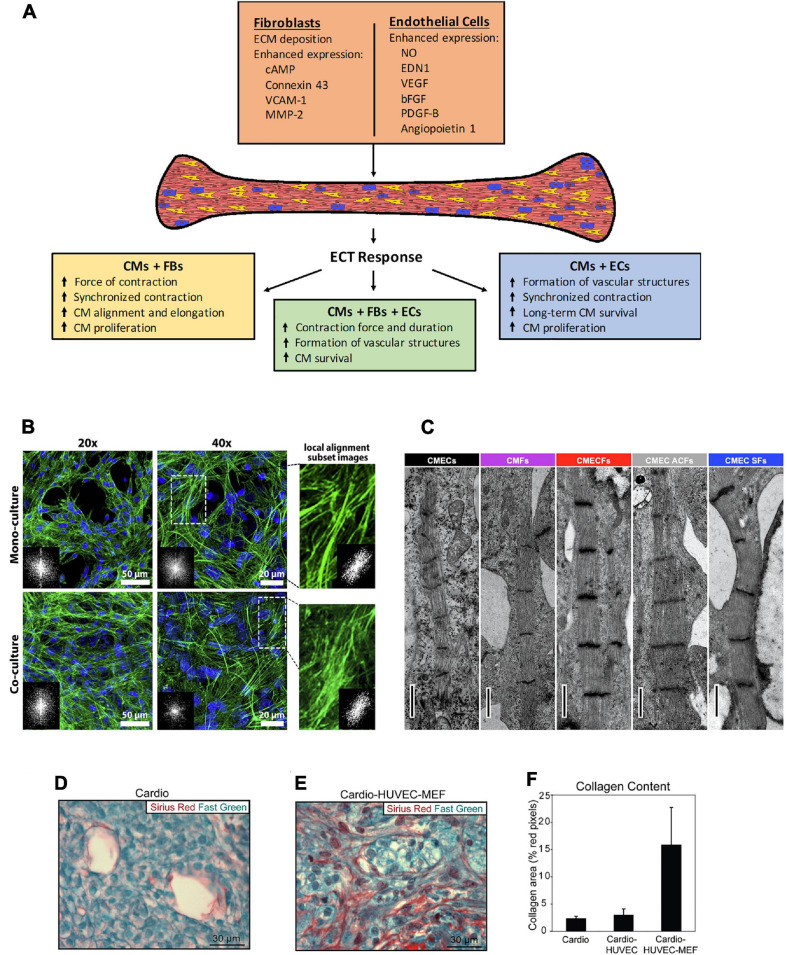
**(A)** Schematic of the effects observed in engineered cardiac tissues fabricated with the inclusion of non-CMs, and the potential mechanisms involved. CMs, cardiomyocytes, FBs, fibroblasts, ECs, endothelial cells. **(B–E)** Histological representation of effects from co-culture in various types of 3D microenvironment. **(B)** The cytoskeleton organization and analysis of F-actin fiber alignment within PNJ-Gelatin hydrogel 3D microenvironment. F-actin fibers (green) stained images in monoculture of neonatal rat ventricular CMs and co-culture of CMs-CFs (2:1 ratio). Both culture groups representing the cytoskeleton organization at 20 and 40 magnifications; FFT images (inset) indicate fiber alignment within the formed 3D cardiac tissue. The magnified spots and related inset FFT images illustrate the local alignment of F-actin fibers. **(C)** Cardiac fibroblasts promote structural maturation of hiPSC-CMs in microtissues. Representative transmission electron microscopy images showing sarcomeres in different microtissues. Scale bar: 1 mm. Cellular composition of cardiac scaffold free 3-D microtissues as follows – CMECS: 85% hiPSC-CMs +15% hiPSC-ECs; CMFs: 85% hiPSC-CMs +15% hiPSC-CFs; CMECFs: 70% hiPSC-CMs +15% hiPSC-ECs+15% hiPSC-CFs; CMEC ACFs: 70% hiPSC-CMs +15% hiPSC-ECs+15% human adult cardiac fibroblasts (ACFs); CMEC SFs: 70% hiPSC-CMs +15% hiPSC-ECs+15% skin fibroblasts (SFs). **(D–F)** “Tri-cell” cardiac patches containing hESC-derived cardiomyocytes, HUVECs, and mouse embryonic fibroblasts (MEFs) in 1:1:0.5 ratios, respectively; (cardio-HUVEC-MEF patches) had more collagen fibrils compared with cardio-only and cardio-HUVEC patches. Patch sections were stained by using Sirius red (collagen) and fast green (other tissue elements). Representative cardio-only **(D)** and cardio-HUVEC-MEF **(E)** patches. **(F)** Cardio-HUVEC-MEF patches had greater than fivefold collagen per unit area than cardio-only or cardio-HUVEC patches. Adapted and reprinted with permission from **(B)** (Navaei et al., 2016), **(C)** (Giacomelli et al., 2020, doi: 10.1016/j.stem.2020.05.004, https://creativecommons.org/licenses/by/4.0/), and **(D–F)** (Stevens et al., 2009).

While most therapies aimed at improving the contractile function of the heart focus on the CMs, the role of nonmyocytes is not negligible and requires further study, with better understanding of their functional interplay (Tian and Morrisey, 2012; Gambardella et al., 2017). Advancing the maturation of ECTs is an ongoing area of research. Consequently, multiple studies have investigated the effect of CM co-culture with different nonmyocyte cell types. In this review, we explore the contribution of nonmyocytes in cardiac tissue engineering (Figure 1A).

## Fibroblasts

Cardiac fibroblasts (FBs) play an important role in extracellular matrix (ECM) modulation and also have an effect on cardiac function; cardiac FB–CM heterocellular coupling influences electrical conduction in the heart; gap junctions and intercellular calcium signaling participate in this cell–cell communication (Doppler et al., 2017). In multiple studies, the inclusion of FBs in tissue fabrication improved electrophysiological properties of engineered tissues (Radisic et al., 2008; Liau et al., 2011; Matsuura et al., 2011; Saini et al., 2015; Iwamiya et al., 2016; Navaei et al., 2016; Ronaldson-Bouchard et al., 2018; Beauchamp et al., 2020). Cardiac spheroids with human embryonic cardiac FBs constructed by Beauchamp et al. (2020) demonstrated higher rate of contraction compared to spheroids with only hiPSC-CMs; adding the fibroblasts did not induce any arrythmogenic effects. Co-culture of fibroblast with CMs results in higher synchronous tissue contractions compared to tissues with only CMs (Matsuura et al., 2011; Iwamiya et al., 2016; Navaei et al., 2016). Navaei et al. (2016) found denser and more uniform organization of F-actin fibers in co-culture tissues that exhibited higher synchronous contraction, suggesting that cytosketelon organization may play a role in promoting synchronicity (Figure 1B). Radisic et al. (2008) reported higher amplitude of contraction in tissues from both concurrent neonatal rat CM-FB co-culture and tissues pre-treated with fibroblasts; for pre-treatment, fibroblasts were cultured on the scaffold prior to the addition of CMs; the pre-treatment group displayed lower excitation threshold and higher amplitude of contractions, out-performing CM-only ECTs and ECTs made from concurrent culture of cardiomycoytes and fibroblasts. Futhermore, fibroblast co-culture enhances structural maturation of ECTs. CMs become elongated and exhibit better alignment and cell-based network formation in co-culture (Nichol et al., 2008; Radisic et al., 2008; Saini et al., 2015; Beauchamp et al., 2020). In addition to improving morphology, Nichol et al. (2008) observed 53% less neonatal rat CM apoptosis, and Iwamiya et al. (2016) found a substantial increase in mouse embryonic stem cell (ESC)-derived CM proliferation resulting from the inclusion of fibroblasts in tissue fabrication. Fibroblast co-culture enhances the expression of cardiac markers in CMs (Radisic et al., 2008; Matsuura et al., 2011; Saini et al., 2015; Navaei et al., 2016; Li et al., 2017; Ronaldson-Bouchard et al., 2018). Despite the noted benefits of fibroblasts in ECTs, the source and the age of fibroblast play an important role on how fibroblasts affect the tissues. Li et al. (2017) found that tissues with adult murine fibroblasts displayed slower conduction velocity, higher stiffness, and decreased calcium transient amplitude compared to those with murine fetal fibroblasts; in this case, the adult fibroblasts induced pro-fibrotic effects on the engineered tissue. Indeed, fibroblasts play a role in pathologic fibrotic remodeling of the heart, and they have been included in ECT to model cardiac fibrosis, with the addition of TGFβ as a trigger for fibrosis (Sadeghi et al., 2017; Mastikhina et al., 2020) and by modulating the CM:FB ratio, where 3:1 models normal myocardium and 1:3 is fibrotic (Wang et al., 2019).

## Endothelial Cells

Through intercellular communication, ECs, among other functions, influence cardiac performance and remodeling; small molecules and peptides secreted by ECs play a critical role in CM contractility (Brutsaert, 2003; Segers et al., 2018; Talman and Kivelä, 2018). One of the goals of EC co-culture in tissue engineering is to create vasculature. ECs are capable of forming capillary-like structures in ECTs when co-cultured with CMs (Narmoneva et al., 2004; Sekine et al., 2008; Garzoni et al., 2009). These structures enhance CM organization and support long-term survival in engineered tissues (Narmoneva et al., 2004; Garzoni et al., 2009). Sekine et al. (2008) reported the formation of greater number of capillaries in EC co-culture tissues compared to neonatal rat CM-only; after transplantation onto infarcted rat heart, capillaries from the tissues spread into host heart and connected with host capillaries, this was accompanied by recovery of cardiac function, documented by improvement in fractional shortening and reduced fibrosis in the surrounding host myocardium; achieving vascularization after engraftment is relevant when aiming to restore blood flow in compromised areas of the ischemic heart. In the presence of vascular structures formed by ECs, co-culture tissues have better contractile properties (Narmoneva et al., 2004; Garzoni et al., 2009). Compared to CM-only tissues and tissues made from simultaneous EC and CM co-culture, Narmoneva et al. (2004) found significantly larger areas of synchronized contraction in tissues containing mouse CMs that were seeded on preformed EC vascular networks. However, there are studies that reported no significant difference in electrophysiological properties such as QT and RR intervals (Giacomelli et al., 2017), force generation, and calcium transients of hPSC-CMs (Burridge et al., 2014) between EC co-culture and hPSC-CM-only tissues. In terms of gene expression, Giacomelli et al. (2017) observed that addition of ECs results in upregulation of genes associated with sarcomere structure, ion channel, and Ca^2+^-handling. Burridge et al. (2014) also reported significant increase in the expression of the voltage-dependent L-type Ca^2+^ ion channel Cav1.2 and higher expression (although not significant) of hESC-CM structural genes. ECs also support maturation of hiPSC-CMs in co-culture tissues through the secretion of endothelin-1 (EDN1) and nitric oxide (NO) (Giacomelli et al., 2020). Furthermore, ECs in ECTs may promote the expression of gap junction protein connexin-43 (Cx43) (Narmoneva et al., 2004; Giacomelli et al., 2020). While Hussain et al. (2013) reported no visible Cx43 in neonatal rat-CM-EC co-culture tissues, using immunostaining, Narmoneva et al. (2004) observed notably higher Cx43 expression in mouse CM-ECs co-culture tissues, indicating the presence of junctions not only between CMs but also between ECs and CMs. The differences in findings may be due to the varying methodology used in these studies. Nevertheless, there is strong evidence to suggest that ECs have a positive impact on CM survival and proliferation in co-culture tissues (Narmoneva et al., 2004; Caspi et al., 2007; Tulloch et al., 2011). In co-culture tissues, Caspi et al. (2007) observed a significantly higher percentage of proliferating CMs, shown by immunostaining for Ki67, compared to CM-only tissues. CM proliferation may be stimulated by mitogens derived from ECs, as suggested by findings from Tulloch et al. (2011) where there was an increase in DNA synthesis rates in EC co-culture tissues under all tested conditions: no stress, systolic stress, and cyclic stress.

## Endothelial And Smooth Muscle Cells Co-Culture

Smooth muscle cells (SMCs) play an important role in the heart; they regulate the tone of blood vessels (Brozovich et al., 2016), and at the molecular level, SMCs affect gap junctions and the expression of Cx43 in CM (Zhou et al., 2020). Transplantation of SMC, EC, and CM co-cultures improves heart function after myocardial infarction (MI) in animal models (Ye et al., 2015; Gao et al., 2018). Gao et al. (2018) found exomes released by tri-culture tissues reduced hiPSC-CM apoptosis; after transplantation of these tissue onto infarcted swine heart, improvements in left ventricular (LV) wall stress, infarct size, and vascular density was observed; these tissues also prevented the reduction in phosphorylation of the sarcomere proteins ENH2 and cTnI after MI. Reduced phosphorylation of sarcomere proteins may be induced by MI (Peng et al., 2014), and it correlates with poor contractility (van der Velden et al., 2004); therefore, stopping phosphorylation reduction may improve heart function after MI. Stromal cells, which have the potential to differentiate into SMCs (Vater et al., 2011), also show enhancement in tissue properties when co-cultured with ECs and CMs (Kreutziger et al., 2011; Tulloch et al., 2011; Burridge et al., 2014; Giacomelli et al., 2020). Tulloch et al. (2011) reported significant increase in vascular structure formation after the addition of human marrow stromal cells in EC-hESC-CM co-culture tissues. Similarly, Kreutziger et al. (2011) observed vessel formation after transplantation of stromal cell co-culture tissue onto uninjured rat heart; stromal cells in these tissues produce ECM components such as fibrillar collagen, hyaluronan, and versican. Furthermore, studies involving co-culture of mural cells show positive impact on vessel formation (Caspi et al., 2007; Masumoto et al., 2016). Caspi et al. (2007) found α-smooth muscle actin positive cells derived from embryonic mouse fibroblasts that integrated into the blood vessels in the tissues. These findings indicate that stromal cells play a crucial role in the formation of vasculature in ECTs. In addition to the formation of graft-derived vasculature after tissue engraftment onto rat heart, Masumoto et al. (2016) found indications of hiPSC-CM sarcomere maturation and better alignment facilitated by mural cells. For translational purposes, Ishigami et al. (2018) produced clinical-scaled engineered cardiac sheets composed of iPSC-CM, EC, and mural cells; these were transplanted onto the heart of mini-pigs after myocardial infarction, resulting in functional and structural improvements. Since all mentioned studies include ECs in the co-culture tissues, the role of SMCs in the observed tissue improvements requires further investigation.

## Fibroblast and Endothelial Cell Co-Culture

As previously described, tissues from CM-EC and CM-FB co-cultures have better properties than CM-only tissues. Here, we discuss the impact of CM-FB-EC co-cultures in ECTs. Compared to tissues with neither FBs nor ECs, tri-culture tissues have enhanced contractile function (Naito et al., 2006; Giacomelli et al., 2020). Giacomelli et al. (2020) observed longer contraction duration and higher contraction amplitude in tri-culture tissues; along with electrophysiological maturation, tri-culture tissues also displayed sarcomeric maturation relative to hiPSC-derived CM-EC and CM-FB co-cultures (Figure 1C). Multiple studies revealed that tri-culture enhances the formation of vessel-like structures (Naito et al., 2006; Caspi et al., 2007; Stevens et al., 2009; Lesman et al., 2010; Kreutziger et al., 2011; Tulloch et al., 2011). Higher level of vasculature in tri-cultures suggests ECs promote vasculature regardless of the inclusion of FBs. Vasculature formation may improve ECT features. Stevens et al. (2009) created tissues from co-culture of hESC-CMs with human umbilical vein endothelial cells (HUVECs) and mouse embryonic fibroblasts (MEFs) that developed significantly more vessel structures and collagen content (Figures 1D–F) than CM-only tissues; these tri-culture tissues displayed passive mechanical properties with greater resemblance to native myocardium (neonatal pig and rat cardiac tissue), and transplantation onto naïve rat heart resulted in improved CM survival and larger graft formation. The vessels in tri-culture tissues can function after transplantation and are perfused by host circulation (Stevens et al., 2009; Lesman et al., 2010). Furthermore, the presence of FBs in the tri-culture improves EC survival and proliferation (Caspi et al., 2007). Despite the noted benefits of including FBs and ECs in ECTs, the methods and cellular composition used to create co-culture tissues may affect ECT features. For example, Iyer et al. (2009) constructed tissues from simultaneous co-culture of murine CMs, ECs, and FBs that failed to exhibit contractile activity, while the CM-only tissues displayed superior functions; however, CMs seeded on preformed networks of ECs and FBs resulted in contractile tissues.

## Mechanisms

The co-culture of nonmyocytes and CMs in ECTs enhanced many tissue properties (Table 1). Here, we describe potential mechanisms behind the observed tissue improvements (Figure 1A). As discussed earlier, multiple studies reported better electrophysiological properties in tissues containing FBs compared to CM-only tissues. In FB-CM tissues, enhanced contractility may be due to a higher expression of Cx43, which allows ions and solutes to pass between cells (Saini et al., 2015). In addition to increased Cx43 expression, FBs support maturation of CMs via cAMP (cyclic adenosine monophosphate) signaling (Giacomelli et al., 2020). FBs also promote CM proliferation; Iwamiya et al. (2016) found that mouse neonatal cardiac FBs in ECTs expressed vascular cell adhesion molecule-1 (VCAM-1), and treatment of mouse ESC- derived CMs with VCAM-1 increased CM population in monoculture; therefore, it is likely that FBs enhance CM proliferation through VCAM-1 signaling. Furthermore, FBs affect CM morphology in ECTs via matrix metalloprotease-2 (MMP-2) expression; Nichol et al. (2008) observed significant higher expression of MMP-2 in FB co-culture tissues that exhibited greater neonatal rat CM alignment, and inhibition of MMP-2 eliminated the increased alignment in co-culture tissues.

**TABLE 1:** Summary of the effects of non-cardiomyocytes on engineered cardiac tissues.

**Cell composition**	**CM + Fib + EC**	**CM + Fib**	**CM + EC**
Increased contractile force	X (Giacomelli et al., 2020)	X (Radisic et al., 2008; Liau et al., 2011; Ronaldson-Bouchard et al., 2018)	X (Masumoto et al., 2016)
Enhanced alignment and sarcomeric banding	X (Iyer et al., 2009; Lesman et al., 2010; Giacomelli et al., 2020)^a^	X (Nichol et al., 2008; Radisic et al., 2008; Liau et al., 2011; Saini et al., 2015; Iwamiya et al., 2016; Navaei et al., 2016; Ronaldson-Bouchard et al., 2018)^b^	X (Narmoneva et al., 2004; Masumoto et al., 2016)
Upregulation of maturation genes	X (Caspi et al., 2007)		X (Masumoto et al., 2016)
Promote electrical maturation	X (Giacomelli et al., 2020)		
Increase synchronicity		X (Liau et al., 2011; Hussain et al., 2013; Saini et al., 2015; Navaei et al., 2016)	X (Narmoneva et al., 2004)
Increase conduction velocity		X (Liau et al., 2011)	
Faster spontaneous beat rate		X (Liau et al., 2011; Saini et al., 2015)^c^	
Promote vascular network formation	X (Caspi et al., 2007; Stevens et al., 2009; Lesman et al., 2010; Kreutziger et al., 2011; Tulloch et al., 2011)^d^		X (Narmoneva et al., 2004; Sekine et al., 2008; Garzoni et al., 2009; Tulloch et al., 2011)
Increase CM survival	X (Stevens et al., 2009)		X (Narmoneva et al., 2004)
Increase CM proliferation	X (Caspi et al., 2007)	X (Iwamiya et al., 2016)	X (Caspi et al., 2007; Tulloch et al., 2011)

*CM, cardiomyocytes, Fib, fibroblasts, EC, endothelial cells. ^*a*^Effect observed in pre-culture, but not in co-culture (Iyer et al., 2009). ^*b*^Effect observed with the addition of cardiac fibroblasts, but not with dermal fibroblasts (Iwamiya et al., 2016). ^*c*^Increase by culture day 5, with gradual decrease to 0 Hz by day 14 (Saini et al., 2015). ^*d*^This includes stromal cells in addition to CM + Fib + EC (Kreutziger et al., 2011; Tulloch et al., 2011).*

A notable feature of EC co-culture tissues is the presence of vascular networks. ECs form blood vessels through angiogenesis; in spheroids, angiogenic sprouting is stimulated by vascular endothelial growth factor (VEGF) (Garzoni et al., 2009). Increased vasculature formation in co-culture correlates with the greater production of VEGF, basic fibroblast growth factor (bFGF), and hepatocyte growth factor (HGF) (Sekine et al., 2008). Caspi et al. (2007) found that addition of mouse embryonic fibroblasts significantly increases endothelial vessel network formation, along with the expression of VEGF, platelet-derived growth factor (PDGF)-B, and angiopoietin 1 (Ang-1); VEGF may also be responsible for the enhanced EC viability observed in co-culture tissues. Furthermore, a study with neonatal rat ventricular myocytes in 2D cell culture demonstrated that VEGF promotes the expression of Cx43 (Pimentel et al., 2002). However, Narmoneva et al. (2004) observed no change in apoptosis or Cx43 expression when ECs and mouse CMs were co-cultured with and without neutralizing VEGF antibody. Although the differences in findings may be due to the distinct methods used in these studies, it suggests that VEGF-related mechanism in ECTs requires further investigation.

Two methods of engineered tissue culture, (1) concurrent co-culture and (2) CMs seeded on pre-culture of nonmyocytes (pre-culture or pre-treatment), with similar cellular composition result in strikingly different ECTs. Compared to concurrent co-culture, the pre-culture group exhibited better contractile properties, along with enhancement of other features, in tissues with FBs (Radisic et al., 2008), ECs (Narmoneva et al., 2004), or both (Iyer et al., 2009). Pre-culture of the nonmyocyte cells before seeding CMs may have allowed them to deposit greater ECM components, enabling better CM attachment and survival (Radisic et al., 2008; Iyer et al., 2009). Radisic et al. (2008) found increased expression of prolyl-4-hydroxylase, which plays a crucial role in collagen synthesis (Pihlajaniemi et al., 1991), in pre-culture group with FBs, suggesting enhanced collagen deposition by FBs in pre-culture tissues. The favorable environment created by the pre-culture of nonmyocytes through ECM deposition likely improved tissue function in these studies.

## Summary and Perspectives

While there is evidence of beneficial effects from including non-CMs in the cellular mix of ECT fabrication; in some instances, non-CMs were not beneficial or contributed to model disease. The heterogeneity of results points to the need to further investigate cardiac cell–cell interactions to achieve the ideal ECT of multicellular composition that reliably mimics the native myocardium. Our perspective is the fabrication ECT containing iPSC-CM and iPSC derived nonmyocytes (FBs, ECs, and SMCs) at different ratios; maintaining the iPSC-CM at 70% or more of the cellular composition (Sekine et al., 2008; Giacomelli et al., 2017, 2020; Wang et al., 2019). Investigating different cell compositions will allow identification of the ratio needed to yield ECTs with optimal structure and function. Eventually, these cell mixtures could be tested in the development of bio-inks for 3D printing (de Santis et al., 2021). Additional strategies include treatment of the nonmyocyte population to harness their potential benefits. For example, treatment of fibroblasts with TGFβ inhibitors to prevent their transition into myofibroblast (Figtree et al., 2017; Cáceres et al., 2018), and thus avoid the pathologic fibrotic phenotype. Also, cell preconditioning can be applied to exploit their autocrine effects; Borosch et al. (2017) found that extracellular vesicles derived from hypoxia-treated fibroblasts favored cell migration and significantly enhanced scratch area reduction. Fabrication of ECT with a diverse combination of hiPSC-derived cell types provides a platform to systematically interrogate human cell–cell crosstalk and advance our understanding of cell–cell interaction in the human heart. The application of ECT in the clinical setting requires further pre-clinical testing in large animal models, overcoming challenges for the fabrication of larger engineered tissues while maintaining cell survival for engraftment and enhanced function.

## Conclusion

Engineered cardiac tissue have translational potential which can be broadly divided into two applications. The first is as surrogate of human myocardium that may serve as a platform for modeling heart disease and investigate novel therapies; for this end, the use of iPSCs furthermore provides the opportunity to apply the ECT for personalized medicine. The second, for transplant, is to repair damaged myocardium. Every effort to produce ECTs that better represent the native myocardium will be beneficial to both applications.

Studies show nonmyocytes contribute to structural and functional maturity of ECTs. However, the studies discussed in this review used an extensive variety of cell types and composition. They also collected a wide range of data, thus making it difficult to find multiple studies with similar methodologies and objectives. Studies often report the observed effect of nonmyocytes on engineered tissues without experimentally identifying the mechanisms. Therefore, the mechanisms behind the interactions between non-CMs and CMs in engineered tissue remain largely unexplored. Further investigations with CMs and nonmyocyte cells from a single source would reduce the variability in methods and help reveal mechanisms that may aid in the development of better tissues and therapies for cardiovascular disease.

## Author Contributions

Both authors contributed to the article and approved the submitted version.

## Conflict of Interest

The authors declare that the research was conducted in the absence of any commercial or financial relationships that could be construed as a potential conflict of interest.
